# Differential involvement of the senses in disgust memories

**DOI:** 10.1098/rsos.231156

**Published:** 2024-03-20

**Authors:** Elliott Lamond, Supreet Saluja, Chloe Hislop, Richard J. Stevenson

**Affiliations:** ^1^ Department of Psychology, Macquarie University, Sydney, New South Wales 2109, Australia; ^2^ Department of Clinical Neuroscience, Karolinska Universitet, Stockholm 171 76, Sweden

**Keywords:** smell, taste, touch, disgust, memories, natural experiences

## Abstract

One prediction derived from the disease avoidance account of disgust is that proximal disgust cues (smells, tastes and touches) should elicit this emotion more intensely than distal disgust cues (sights and sounds). If correct, then memories of disgusting experiences should involve smelling, tasting or touching to a greater degree than seeing or hearing. Two surveys were conducted on university students to test this idea, drawing upon their naturalistic experiences. Survey 1 (*N* = 127) asked participants to detail their most *memorable* disgusting, fear-provoking, morally repulsive and yucky/gross experience, with each recollection self-rated for sensory involvement. Survey 2 (*N* = 89) employed the same task, but this time, participants recollected their most common disgusting, fear-provoking, morally repulsive and yucky/gross experience in the preceding week. The majority of disgusting experiences were core disgusts—i.e. related to disease/pathogen presence or stimuli. The proximal and distal sensory cues contributed equally to individuals’ most memorable core disgust experiences, but the proximal senses were more involved than the distal senses in individuals’ most common core disgust experiences. Further, the proximal sensory cues, as compared with the distal sensory cues, were signficantly more involved in core disgust experiences than in morally repulsive and fear-provoking experiences. The implications of these findings for a disease avoidance account of disgust, for multi-sensory disgust research, and core disgust’s classification as an emotion or a drive, are discussed.

## 1. Introduction

The main function of the basic emotion of disgust is to prevent disease and toxins from entering the body [1–4]. Several hypotheses related to disgust’s role in disease avoidance have been generated and tested (e.g. source effects, disgust elicitor–disease relationships and disease-related stigmatization [5,6]). However, one hypothesis remains largely untested. Proximal sensory disgust cues—i.e. involving smell, taste and touch—are suggested to result in more intense disgust experiences than distal sensory disgust cues—i.e. involving vision and audition [7–11]. It has been reasoned that proximal sensory cues are more intense elicitors of disgust because pathogens and toxins enter the body at surfaces the proximal senses bind to—i.e. nasal passageways (smells), mouth (tastes and smells) and skin openings (touch). In contrast, sights and sounds often signal distant potential harms, thereby posing a less immediate threat than proximal sensory disgusts [7,9–11]. If proximal sensory cues evoke more intense disgust than distal sensory cues, memories of disgusting experiences should feature smelling, tasting and touching to a greater degree than seeing or hearing. Thus, the primary aim of the two surveys reported here was to test this hypothesis.

The initial claim that some senses may elicit disgust more intensely than others seems to have been made by [8]. He proposed that disgust was most strongly evoked by taste, followed by smell, touch, sight and sound. The centrality of taste to disgust is generally agreed on, with the distaste reflex—i.e. expulsion of bitter, usually toxin-related tastes from the mouth—said to represent the evolutionary origin of this emotion [12,13]. As smells can signal danger both distally (i.e. via orthonasal olfaction) and proximally (i.e. via retronasal olfaction), olfaction is theorized to be both pertinent to harm detection and avoidance at a distance (e.g. distal—smelling rotten odours in the environment) when food is placed in the mouth (e.g. proximal—smelling rotten odours in the mouth [10,11,14]). For touch—which occurs both actively (touching) and passively (being touched)—this proximal sense is important for localizing things to the surface of the body and, relatedly, to contamination (i.e. the ability of any neutral object to be rendered disgusting via contact with a disgust elicitor [15–17]). For the distal senses (i.e. vision and audition), these are suggested to be more important for the anticipation of disgust—i.e. imagining contact with something revolting [8,10,11]. So, while vision and audition should evoke disgust to avoid closer contact with an elicitor, closer contact itself, registered by the proximal senses, should evoke far greater disgust because of the nearness of the pathogen/toxin threat and hence the need for a more rapid reaction.

Another reason that proximal disgust cues may evoke more intense responses is that disgust to them may be hard-wired—i.e. conserved across species and development. For taste, the distaste reflex is seen in human neonates (only a few hours old), non-human primates (i.e. New World and Old World monkeys) and many other mammals (e.g. rats, cats and ferrets)—indicating that the detection of bitter/toxin related threats may be present with minimal learning [12,18,19]. Similar to taste, olfactory cues that trigger disgust may be evolutionary salient cues of disease and toxin presence. Three low-molecular-weight compounds that signal putrefaction (i.e. nitrogen-containing thiols, sulphur-containing indoles and short-chain fatty acids) are found in most malodours (i.e. foot, breath, mouth malodours, vomit and faeces) and are detected by humans and other species (e.g. magpies, cats, rats and New World and Old World monkeys) at very low thresholds [20]. For touch, Sarabian *et al*. [21] found that chimpanzees (*Pan troglodytes*) were significantly less likely to eat when their food was placed on a wet soft object (disease-related tactile properties) than on a hard dry object, providing some preliminary support that the detection and avoidance of disease-relevant tactile cues are conserved in primates. Taken together, proximal disgust cues may evoke more intense disgust responses than distal disgust cues because they may be hard-wired.

Notwithstanding the material presented above, there is also some evidence that runs contrary to the hypothesis that proximal sensory disgust cues evoke a stronger response than distal cues. An experimental investigation of disgust elicitation across four of the five senses (excluding taste) found that disgusting sights and sounds were the most powerful elicitors, whereas smells and touch-related objects were reported as less disgusting [7]. This was the first study to compare disgust responses with the same object across modalities, and the findings would suggest that the distal senses are, in fact, *more potent* than the proximal senses in eliciting disgust.

While laboratory studies have many strengths, they also have some limitations. A key concern is that participants in laboratory studies probably know that the stimuli are harmless. Critically, this has different implications for the distal and proximal senses. For vision and audition, harmlessness is irrelevant, because even when you know the depicted stimuli are not physically present, they lose none of their forces as elicitors (e.g. colour pictures of gore and sound of vomiting). In contrast, olfactory and tactile stimuli need the physical presence of the actual disgust elicitor to be perceived as a threat. Participants would be unlikely to believe that they are really touching or smelling a disgust elicitor. For taste, it would not be ethical to tell a participant that they are consuming disease-related stimuli (core disgust elicitors)—and indeed, it was for this reason that the assessment of taste disgust was omitted in the study by Croy *et al*. [7]. The consequence of this may be to amplify visual and auditory disgust at the expense of smell, touch and taste. One way to address this issue, without raising ethical concerns, is to examine naturally occurring instances of disgust (i.e. memories of disgusting experiences). If these also demonstrate the same findings as Croy *et al*. [7], this would suggest that there may be no special proximal advantage.

The primary aim of this study was to test if the proximal senses (smell, taste, active touch and passive touch) feature more prominently in disgust elicitation than the distal senses (sight and sound) when people recall their prior disgust experiences. To this end, we asked people to draw upon examples from both their recent and distant past. We used this approach to check if memory biases might affect which senses are considered most relevant in these remembered disgust experiences, as more recent memories may more accurately reflect the relative contribution of the senses than older memories. A second aim was to examine if the proximal senses feature more prominently than the distal senses in core disgust experiences (disgust related to disease/toxin presence), relative to (i) other forms of disgust (moral disgust experiences) and (ii) other negative emotions (fear experiences). We used moral disgust (i.e. disgust directed at moral/ethical violations) because this is distinct from core disgust (i.e. disgust to toxins/disease [22]) and fear because this is probably the most similar of the basic emotions to disgust [23].

To address these aims, participants completed two surveys spaced one week apart. Survey 1 was cross-sectional. Participants were asked to detail their most memorable disgusting, fear-provoking, morally repulsive (moral disgust) and yucky/gross (core disgust) experiences. Consistent with Asch’s [24] impression formation approach, participants were asked to describe these experiences in their own words, with minimal guidance as to what they should or should not report. Next, participants’ experiences were presented back to them, and they were asked to rate if and how much each sense contributed to the emotional experience. This is our key measure to assess whether the proximal sensory cues are involved to a greater degree than the distal sensory cues. We chose to ask in this manner because it did not presume what aspect of the sensory cues resulted in greater disgust (e.g. proximal sensory stimuli may be more intense, attention to the proximal cue could be stronger or the quality of the disgust experience could be different).

After completing survey 1, the same participants were sent an online Google document. They were asked to log every day, for six consecutive days, any disgusting, fear-provoking, morally repulsive and yucky/gross things that they came across. The Google document functioned solely as an aid to memory. After completing their week of self-data collection, participants were sent the second survey. Here, participants detailed their past week’s most common disgusting, fear-provoking, morally repulsive and yucky/gross experiences and the involvement of each sense (as for survey 1) in these experiences.

## 2. Methods

### 2.1. Participants

One hundred and twenty-seven participants completed the first survey (87 females, 38 males and 2 reported others; age, *M* = 20.8, s.d. = 6.3) and 90 completed the second survey (60 females, 28 males and 2 others; age, *M* = 21.4, s.d. = 7.2). The sample size was based on effect sizes derived from past survey studies examining disgust experiences (i.e. Cohen’s *d* values ranging from 0.3 to 0.4, namely, small to medium effect sizes [25,26]). All participants were from Macquarie University and received course credit for taking part. Participants were initially informed that the study assessed memories and experiences concerning emotion in everyday life. Once the study was completed, they were debriefed and told that it was specifically examining the role of the senses in disgust experiences. The study protocol was approved by the Macquarie University Human Research Ethics Committee (520221126136899). All de-identified data are accessible online on Dryad.

### 2.2. Materials

A 32-item self-report questionnaire developed by Haidt *et al*. [2] was used to measure individual differences in disgust sensitivity. This measure was selected as it is the only disgust survey to be behaviourally validated [27]. Scores could range from 0 to 32, with higher scores indicating higher disgust sensitivity. The internal consistency for the Disgust Scale in the current study was moderate to strong (*α* = 0.80), and this is comparable to past studies (*α* = 0.84 [2]). Only the total score was used.

The Perceived Vulnerability to Disease (PVD) is a 15-item self-report questionnaire developed by Duncan *et al*. [28], which measures an individual’s perceived vulnerability to infection and their discomfort when being exposed to potentially infectious situations. PVD scores can range from 15 to 105, with higher scores indicating greater PVD. In the current study, the PVD showed moderate to strong internal consistency (*α* = 0.79), and this is comparable to past studies (*α* = 0.82 [28]). Only the total PVD score was analysed.

### 2.3. Procedure

**Figure 1.**
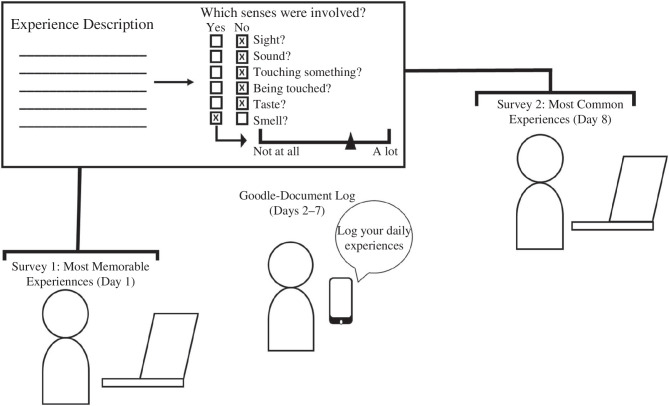
Summary of the experimental procedure. In survey 1, participants reported their most memorable disgusting, fear-provoking, morally repulsive and yucky/gross experiences, and then rated how much each sense contributed to them. After completing survey 1, participants logged the disgusting, fear-provoking, morally repulsive and yucky/gross experiences they experienced over the following week. The second survey was completed 7 days after the first and was the same as the first survey but focused on participants’ most common experiences.

This study consisted of two surveys, each completed one week apart (see figure 1). The first survey had five sub-sections presented in the following order. First, participants consented to (or declined to continue with) the study. Second, basic demographics and contact information were collected (i.e. age, gender, phone number and email). Third, participants were asked three questions pertaining to the emotion of disgust: (1) to detail what was the most memorable ‘disgusting thing’ they had ever experienced in their life (via an open-ended question); (2) their age during this experience; and (3) how disgusting this experience was (ranging from 0 ‘not at all’ to ‘100’ very). Fourth, participants were asked these same three questions again—now, pertaining to three other emotional states: fear, moral repulsion and yucky/gross, presented in random order. Fifth, using a carry-forward design, participants’ most memorable emotional experience for each category (i.e. disgust, fear, moral repulsion and yucky/gross) was presented back to them—and for each category, they were asked whether (i.e. yes/no) a sensory modality (i.e. seeing, hearing, smelling, tasting, touching and being contacted by something) was present in their experience (e.g. for seeing, ‘*did you see something in this experience?*’). If they responded ‘yes’, they were asked how much that sense had contributed to their effect on the experience (on a 100-point line scale).

Within 24 h of completing the first survey, participants were emailed their own online Google document. Participants were asked to log anything they naturally came across that was disgusting, fear-provoking, morally repulsive and yucky/gross over the following 6 days. To ensure that participants remembered to log their experiences, three reminder text messages were sent to them over this period (i.e. one message every 48 h).

After logging their experiences for a week, participants were sent the second survey that they were instructed to complete within 48 h of receiving the link. The second survey had five sub-sections presented in the following order. First, participants were asked to refer to their log book (Google document) and detail the most common disgusting experience they had that week. Next, participants were asked how often the experience occurred (via a 5-point category scale; ranging from once a week to more than once daily) and how disgusting the experience was (on a 100-point line scale; ranging from 0 ‘not at all’ to 100 ‘very’). Third, participants repeated step 1 with the three remaining emotional states (i.e. fear, morally repulsive and yucky/gross—presented in a random order). Fourth, participants’ reported experiences were presented back to them, and they were asked if (yes/no) and to what extent (via a 100-point linear scale) each sense (i.e. seeing, hearing, smelling, tasting, touching and being contacted by something) contributed to their experience. Fifth, participants were asked to complete the Disgust Scale [2] and the PVD scale [28]. The Disgust Scale [2] and the PVD scale [28] were given at the end of the second survey, so as not to bias participants on the type of disgust experiences they could have experienced during the week. Following the completion of these scales, participants were debriefed.

## 3. Analyses

### 3.1. Judge-coding procedure and analyses

Judges were asked to code the disgust experiences from surveys 1 and 2, so as to determine what types of disgusts (i.e. core and moral) were reported in the disgust memories and if the experiences were real (as opposed to imagined/media-based). Two judges who were blind to the study aims were asked to read the participants’ most memorable (from survey 1) and most common (from survey 2) disgust experiences and code them on *Format-experienced* and *Type of disgust*. For *Format-experienced*, the judge coded if the recorded event was actually experienced (‘1’ (yes) and ‘0’ (no)), imagined (‘1’ (yes) and ‘0’ (no)) and/or experienced through videos/tv/media (‘1’ (yes) and ‘0’ (no)). For *Type of disgust*, the judge coded if an experience was a core disgust (i.e. disgust directed at anything that is related to disease, poor hygiene, animals/insects that represent disease vectors, sickly appearances, bodily lesions and off foods; ‘1’ (yes) and ‘0’ (no)) and/or a moral disgust (i.e. disgust directed at behaviours, individuals or ideas that violate one’s moral, ethical or social values; ‘1’ (yes) and ‘0’ (no)).

Each judge coded 89/127 of Survey 1’s (most memorable) disgust experiences, with 51 experiences overlapping, and 65/89 of Survey 2’s (most common) disgust experiences, with 37 experiences overlapping. Overlapping stories were used to assess inter-rater agreement. Agreement occurred when both coders had the same value (1/0) for a given sub-category (e.g. core disgust and moral disgust), and disagreement occurred when coders had a different value for a sub-category. As given in table 1, there was nearly perfect agreement between coders across all sub-categories in both surveys. Thus, in the overlapping disgust experiences, each sub-category was scored as present (1) if *at least* one judge coded it as present and absent (0) if neither judge coded it as present.

**Table 1.** Coder agreement by category.

category	sub-category	survey 1—most memorable disgusts	survey 2—most common disgusts
type of disgust	core	51/51 (100%)	37/37 (100%)
	moral	49/51 (96%)	35/37 (95%)
format	imagined	51/51 (100%)	37/37 (100%)
	video	49/51 (96%)	37/37 (100%)
	actual	48/51 (94%)	36/37 (97%)

### 3.2. General analysis

All the data from both surveys were analysed using SPSS v. 26, with graphs prepared in R version 4.2.0. As all the data were non-normal (standardized skewness and kurtosis greater than 3), non-parametric tests were used. Each participant was asked to provide a single memory for a given emotion (disgust, fear, moral repulsion and yucky/gross), meaning the number of participants for a given emotion equates to the number of descriptions analysed. In survey 1 (most memorable experiences), one participant did not report having any fear experience, and seven did not have any morally repulsive experiences, leaving 126/127 and 120/127 participants’ data available for analyses. In survey 2 (most common experiences), one participant did not report having any experiences, leaving 89/90 participants’ data available for analyses. Disgust sensitivity and PVD data were just used to establish the typicality of participants—with average disgust sensitivity (*M* = 21.2; s.d. = 4.4) and PVD scores (*M* = 59.8; s.d. = 11.9) comparable to past samples [29].

There were two preliminary steps before addressing the primary and secondary aims. First, for each participant’s disgust experience, we identified which proximal sense (smell, taste, passive-touch or active-touch) was most involved in their experience (i.e. which had the largest sensory involvement score) and termed this the ‘proximal sensory maximum score for disgust’. Second, for each participant’s disgust experience, we identified which distal sense (seeing or hearing) was the most involved in their experience (i.e. had the largest sensory involvement score) and termed this the ‘distal sensory maximum score for disgust’. This then allows for a contrast of the most intense proximal sense involved in elicitation (which may vary from experience to experience) with the most intense distal sense involved in elicitation (which may also vary from experience to experience). We used maximum instead of aggregate scores, as this seemed to be the best way to answer the primary aim of the study (i.e. do the proximal senses feature more prominently than the distal senses in recollections of disgusting experiences), namely, aggregate scores would have been weighted differently for proximal and distal senses—i.e. there were four proximal senses but only two distal senses—leading to a bias.

To test the secondary aim, two preliminary steps were taken. First, we identified which sense, proximal or distal, was most involved in each remaining emotion experience (i.e. fear, moral repulsion and yucky/gross). These were termed the proximal and distal sensory maximum scores for fear, moral repulsion and yucky/gross experiences, respectively. Second, for each emotion (disgust, fear, moral repulsion and yucky/gross), we subtracted the distal maximum score from the proximal maximum score—to get a ‘proximal minus distal maximum score’ for each emotion. We then contrasted these ‘proximal minus distal maximum scores’, to determine if disgust had more proximal relative to distal sensory involvement compared with other emotions.

## 4. Results

### 4.1. Types of disgusts reported

On the main disgust recollection question, core disgusts were featured most prominently. They constituted 86.6% (110/127) of participants’ most memorable disgusts (from survey 1) and 96% (85/89) of participants’ most common disgusts (from survey 2). Only 18.9% (24/127) of the most memorable and 5.6% (5/89) of the most common disgust experiences were judged as moral disgusts.

These disgust recollections had in the main been actually experienced, with 93.7% (119/127) of the most memorable and 96.6% (86/89) of the most common disgust experiences judged as such. Only 6.3% (8/127) of the most memorable and 3.4% (3/89) of the most common disgust experiences were classified as experienced through videos/images. Finally, only 2.4% of the most memorable (i.e. 3/127 participants) and none of the most common disgust experiences were classified as imagined.

Thus, the majority of the disgust experiences—memorable and common—were actual experiences of core (i.e. pathogen-related) disgusts.

### 4.2. The nature of the reported emotional experiences

In both surveys, the average intensity of disgust, fear, morally repulsive and yucky/gross experiences (i.e. ratings indicating how disgusting, scary, morally repulsive and yucky/gross participants’ experiences were, respectively) were all above 60% of the scale total, indicating emotionally intense experiences (see table 2).

**Table 2.** Descriptive statistics.

	survey	scale	*M* (s.d.)
disgust	1 (memorable experiences)	intensity (0–100)	83.6 (18.0)
		age experienced	15.7 (5.2)
	2 (common experiences)	intensity (0–100)	62.0 (24.8)
		frequency (1–5)	2.3 (0.9)
fear	1 (memorable experiences)	intensity (0–100)	85.1 (17.9)
		age experienced	14.8 (5.8)
	2 (common experiences)	intensity (0–100)	67.1 (28.4)
		frequency (1–5)	2.2 (.9)
yucky/gross	1 (memorable experiences)	intensity (0–100)	73.4 (21.5)
		age experienced	15.5 (4.8)
	2 (common experiences)	intensity (0–100)	62.1 (25.1)
		frequency (1–5)	2.2 (0.9)
moral disgust	1 (memorable experiences)	intensity (1–5)	83.0 (16.9)
		age experienced	16.3 (4.3)
	2 (common experiences)	intensity (0–100)	63.0 (32.1)
		frequency (1–5)	2.3 (0.9)

*Note*: Age was the participants’ age during the experience.

In survey 1 (most memorable), participants’ experiences on average occurred when they were 5–6 years younger than their current age (disgust: *M* = 5.1, s.d. = 5.9; yucky/gross: *M* = 5.3, s.d. = 5.7; moral repulsion: *M* = 5.4, s.d. = 7.1; fear: *M* = 6.1, s.d. = 6.4), indicating they were memorable and not recent. In survey 2 (most common), participants’ experiences on average occurred just over a few times a week, indicating that they were common and recent.

Disgust and yucky/gross experiences were generally multi-sensory, with the proximal senses collectively accounting for 54% (survey 1) to 57% (survey 2) of the senses present in disgust experiences, and 59% (survey 1) to 62% (survey 2) of the senses present in yucky/gross experiences. Fear and morally repulsive experiences were primarily experienced via sight and sound—with these distal senses collectively accounting for 78% (survey 1) to 90% (survey 2) of the senses present in morally repulsive experiences; and 73% (survey 1) to 78% (survey 2) of the senses present in fear experiences. Thus, the majority of core disgust experiences (disgust and yucky/gross)—but not morally repulsive or fear experiences—had a proximal sensory cue present (smelling, tasting, being touched or touching something). For a visualization of the senses identified as present in these emotional experiences, see electronic supplementary material, figure S1.

### 4.3. Aim 1: are smelling, tasting or touching involved to a greater extent than seeing or hearing in core disgust experiences?

#### 4.3.1. Survey 1—most memorable disgust experiences

A Wilcoxon test compared the most involved proximal sense with the most involved distal sense. The proximal senses (*M* = 76.2, s.d. = 37.2) had a comparable degree of involvement to the distal senses (*M* = 77.2, s.d. = 31.3; *Z* = 0.66, *p* = 0.511) in participants’ most memorable disgust experiences.

#### 4.3.2. Survey 2—most common disgust experiences

In this case, the proximal senses (*M* = 75.1, s.d. = 33.5) had a significantly greater contribution than the distal senses (*M* = 61.3, s.d. = 35.1; *Z* = 3.29, *p* = 0.001) in participants’ most common disgust experiences.

### 4.4. Aim 2: are smelling, tasting or touching involved to a greater extent than seeing or hearing in core disgust, relative to fear and moral disgust experiences?

Friedman tests indicated that the involvement of the proximal senses relative to the distal senses varied across the most memorable (*χ* ^2^(3) = 100.62, *p* < 0.001) and the most common emotion experiences (*χ* ^2^(3) = 140.42, *p* < 0.001).

**Figure 2.**
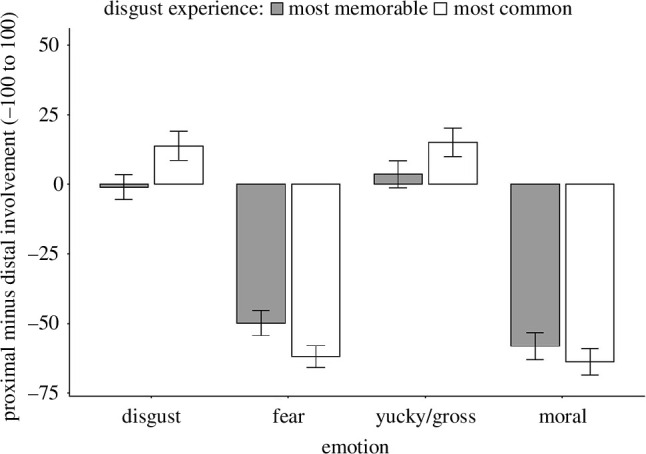
Proximal minus distal sensory involvement by emotion.

Follow-up pair-wise Wilcoxon tests indicated that relative to the distal senses, the proximal senses were significantly more involved in memorable and common disgust and yucky/gross experiences than fear and morally repulsive experiences (all *Z*s > 6.85, *p*s < 0.05/4, family-wise adjusted for emotion). The proximal senses (relative to the distal senses) contributed comparably to common and memorable fear and morally repulsive experiences (all *Z*s < 1.60, *p*s > 0.11). Similarly, the proximal senses (relative to the distal senses) contributed comparably to common and memorable disgust and yucky/gross experiences (all *Z*s < 0.67, *p*s > 0.51; see figure 2).

## 5. Discussion

Since Darwin [8], there has been a suggestion that proximal sensory cues may elicit disgust more intensely than distal cues because disease-related proximal stimuli pose a higher infection risk than disease-related distal stimuli [7,10,11]. We suggested §1 that naturalistic real-world memories should be the best place to look for these effects. In particular, we hypothesized that if smell, taste and touch are more intense elicitors of disgust, the proximate senses should be more involved relative to the distal senses in everyday experiences of core disgust but not in everyday moral disgust or fear (general danger) experiences. Our findings were generally as we had predicted. For core disgust experiences, the involvement of any proximal sense was either comparable to or more intense than the involvement of any distal sense. Furthermore, the proximal senses, relative to the distal senses, were more central to core disgust experiences than to fear or moral disgust.

Before discussing the implications of these findings, it is important to consider their potential limitations. One concern is that the experiences studied here were based on memories. Consequently, differences in the recall and vividness of imagery across the senses may have biased the sensory-involvement ratings, namely, when individuals are asked to recall sensory objects (e.g. a characteristic smell, taste or sight), smells and tastes are rated as the least vivid—the most difficult to imagine and re-experience relative to visual, auditory and tactile objects [30]. Similarly, in their development of the multi-sensory imagery scale, Andrade *et al*. [31] reported that vision and touch were the easiest modalities for people to imagine, with taste and smell being the hardest. Notably, difficulty in imagining chemosensory stimuli seems to occur for memories that are recalled—as in the current study—but not necessarily when memories are evoked by a presented odour (see [32,33]).

The extent to which stored knowledge can be vividly imagined when individuals are asked to recall a memory may differ across the senses owing to modality-specific short-term and executive processes (i.e. pertaining to information retrieval and manipulation [34]). These findings have two implications for our study. The first is that they will generally tend to work *against* demonstrating that the proximal senses are more involved with disgust than the distal senses. The second is that these recollective/imagery effects should apply equally to all of the recollected experiences—disgust, fear and moral disgust—and so differences that emerge *between* emotions should bypass this concern.

One situation where these differences in sensory recall and imagery may be important is in explaining the discrepancy between most-memorable disgusts (proximal equal to distal) and most common disgusts (proximal greater than distal). As the most memorable disgust experiences generally occurred 5–6 years ago, the involvement of the distal senses in these experiences *may* have been easier to recall than the proximal senses. Conversely, recall biases may not have impacted the most common disgust experiences to the same degree, providing a more accurate picture of sensory involvement in disgust.

As we described in §1, the one laboratory study to examine disgust elicitation across the senses found that sights and sounds of disease were more disgusting than touching and smelling disease-related objects [7]. The disparity between our findings (proximal greater than or equal to distal) and Croy *et al*.’s [7] findings (distal greater than proximal) may pertain to methodological differences (i.e. laboratory versus naturalistic study), as we outlined in §1. As laboratory studies require ethical approval and would not allow any harm to come to a participant—something that students are informed of—they may rightly assume that disgust-evoking smells, tastes and touch will carry less disease risk in a laboratory setting than in the real world. Conversely, disgust-evoking sights and sounds may be experienced similarly in the laboratory and real world, as both present little disease risk—i.e. disgusting sights and sounds cannot enter the body and cause sickness. Thus, the disparity between the current study and Croy *et al*.’s [7] findings may reflect the attenuated disease risk of proximal sensory stimuli relative to distal stimuli in laboratory studies. Future studies that measure both psychophysical indices of disgust (i.e. as in [7]) and naturalistic experiences (i.e. as in the current study) are thus needed to provide further insight into the relationship between disgust and senses.

As predicted, the proximal relative to the distal senses were more involved in core disgust experiences than in fear and moral disgust experiences. In line with the disease-avoidance account of disgust, this indicates that smell, taste and touch have a more pronounced role in disease detection and avoidance than general danger avoidance and protection [3,4,35]. An important and unresolved issue that now needs addressing is to understand why the sense–disgust relationship differs between the proximal and distal senses for core disgust cues. As noted in §1, we chose to measure broadly, using a non-specific measure, whether the proximal senses have greater involvement in feelings of disgust—as this allowed us to best address the key aims of the current study—but our data do not offer any direct answer on why this may be the case. We noted in §1 that a proximal cue might elicit disgust more because they are perceptually more intense, might be more attention-demanding or might generate a qualitatively different form of disgust experience (e.g. anticipated versus somatosensory disgust [11]).

Relatedly, while core disgust experiences appeared more multi-sensory—i.e. having multiple senses present—relative to the other emotion experiences (see electronic supplementary material, figure S1), it remains unclear from this study whether there are distinct groupings of the senses for certain types of disgusts. For example, it seems plausible that bodily envelope disgusts (e.g. an ugly wound) may primarily elicit disgust via vision (i.e. unimodal), while food disgusts may be more likely to involve smells, tastes and touch to similar magnitudes (i.e. multi-modal). Future research is thus needed to address if and how the senses may group together for different disgusts identified in the literature [2,4].

Interestingly, moral disgust had a comparable sensory-involvement profile to fear, with both these being different from core disgust. This raises the question of whether core and moral disgust are sub-types of the same emotion. Moral and core disgust have distinctions in their facial [36] and neural [37] profiles. Theoretically, moral disgust may also be more similar to fear than core disgust. Royzman and Sabini [23] proposed that core disgust may be a drive state (akin to thirst and hunger)—which has concrete elicitors and invariant responses that serve to satisfy the drive—whereas moral disgust and fear may both be emotions—which evolved from drive states but that have abstract elicitors and flexible responses. In support of this, while moral disgust (e.g. incest and bullying) and fear (e.g. spiders and shadow on a mammogram) have varied and potentially abstract elicitors—there do not seem to be abstract core disgust elicitors and all seem to be disease/toxin-related [23]. Indeed, these findings merit further investigation into the differences between core and moral disgust to determine their relationship and the structure of disgust.

In conclusion, using a cross-sectional (memorable disgust) and a longitudinal (common disgusts) survey, we find that, in keeping with Darwin’s proposition (1892/2009), the proximal senses feature to a comparable or greater extent than the distal senses, in naturalistic experiences of core disgust. Furthermore, core disgust was predicted more by proximal sensory involvement compared with other affective states (i.e. moral disgust and fear). Finally, moral disgust’s sensory involvement profile paralleled fear, with both differing from core disgust, yet again raising the question of moral disgust’s relationship with core disgust.

## Data Availability

All de-identified data and a read me file, which specifies the analyses, how to conduct them and the variables included in the data file, are available on Dryad [38]. Supplementary material is available online [39].
